# Identification of a Classical Bipartite Nuclear Localization Signal in the *Drosophila* TEA/ATTS Protein Scalloped

**DOI:** 10.1371/journal.pone.0021431

**Published:** 2011-06-23

**Authors:** Adam C. Magico, John B. Bell

**Affiliations:** Department of Biological Sciences, University of Alberta, Edmonton, Alberta, Canada; University of Arkansas for Medical Sciences, United States of America

## Abstract

*Drosophila melanogaster* wing development has been shown to rely on the activity of a complex of two proteins, Scalloped (Sd) and Vestigial (Vg). Within this complex, Sd is known to provide DNA binding though its TEA/ATTS domain, while Vg modulates this binding and provides transcriptional activation through N- and C-terminal activation domains. There is also evidence that Sd is required for the nuclear translocation of Vg. Indeed, a candidate sequence which shows consensus to the bipartite family of nuclear localization signals (NLSs) has been identified within Sd previously, though it is not known if it is functional, or if additional unpredicted signals that mediate nuclear transport exist within the protein. By expressing various enhanced green fluorescent protein (eGFP) tagged constructs within *Drosophila* S2 cells, we demonstrate that this NLS is indeed functional and necessary for the proper nuclear localization of Sd. Additionally, the region containing the NLS is critical for the wildtype function of ectopically expressed Sd, in the context of wing development. Using site-directed mutagenesis, we have identified a group of five amino acids within this NLS which is critical for its function, as well as another group of two which is of lesser importance. Together with data that suggests that this sequence mediates interactions with Importin-α3, we conclude that the identified NLS is likely a classical bipartite signal. Further dissection of Sd has also revealed that a large portion of the C-terminal domain of the protein is required its proper nuclear localization. Finally, a Leptomycin B (LB) sensitive signal which appears to facilitate nuclear export is identified, raising the possibility that Sd also contains a nuclear export signal (NES).

## Introduction

The *Drosophila melanogaster* protein, Scalloped (Sd), is a member of the highly conserved family of TEA/ATTS domain (which is named for the first three identified members of the family, TEF-1, TEC-1, and AbaA, and is hereafter abbreviated as TEAD) containing transcription factors [1]–[3]. This group is represented within a wide range of eukaryotes, ranging from *Saccharomyces cerevisiae* (Transposon Enhancement Control – 1 or TEC-1) to *Homo sapiens* (multiple Transcriptional enhancer factors (TEFs) [4]–[6]). The TEAD is a DNA binding domain [2], [3], however, members of this family are thought to lack transcription activation domains and thus require interactions with transcription intermediary factors (TIFS) to form tissue specific transcription factors [7]–[15]. For instance, *ex vivo* experiments in mouse cells demonstrated that Yes Associated Protein 65 (Yap65 aka Yap1), requires the TEAD containing protein, TEAD-2, for DNA binding and activation of transcription or various reporters. Likewise, TEAD-2 which was not complexed with Yap-65 was unable to activate transcription. Indeed, it was only when the DNA binding domain of TEAD-2 and the transcriptional activating domain of Yap-65 were present in the same complex, that strong reporter activity was seen [11].

Sd also requires at least two TIFs during *Drosophila* wing development. The first is Vestigial (Vg) [9], [10]. Both Sd and Vg are expressed in a similar pattern encompassing the pouch, mesopleura and scutellum in third instar larval wing discs [1], [16]. In order for proper wing blade and margin development to occur, the two proteins must interact to form a transcription factor [9], [10]. Within this complex, both the TEA domain of Sd and the N- and C- terminal domains of Vg are required for activation of transcription [17]–[20]. Furthermore, there is *in vitro* evidence that the binding of Vg to Sd alters the specificity of the TEAD of Sd [21]. The second known wing-expressed TIF of Sd is Yorkie (Yki) [14]. Yki is the downstream effector of the Hippo and Fat pathways in Drosophila, which are involved in regulating cellular proliferation and apoptosis [22], [23]. Yki is known to bind Sd in the developing wing and eye discs, and this binding is required for the proliferation of the respective tissues [14], [24], [25]. Likewise, the Sd/Vg complex has also been implicated in cell survival and proliferation [24], [26]. Outside of the wing, Drosophila myocyte enhancer factor 2 (dMef2) has also recently been identified as a TIF of Sd, implicating Sd in *Drosophila* muscle development, a role the TEAD proteins in mammals and amphibians have long been known to have [15].

In addition to Sd conferring DNA binding ability to Vg and Yki, both of these proteins are largely cytoplasmic when expressed ectopically and thought to rely on signals within Sd for nuclear translocation [9], [10], [14], [25], [27]. The nuclear localization of proteins is mediated by one of two mechanisms. The first is via passive diffusion through the nuclear pore complex (NPC), a mechanism which excludes proteins larger than approximately 40–50 kDa [28], [29]. The second is by an energy-dependent process, an example of which is where proteins containing a nuclear localization signal (NLS) are targeted to the nucleus, via Importin α/β binding and translocation through the NPC (reviewed in [29], [30]).

In *Drosophila*, there are three known members of the Importin-*α* (Imp-*α*) family of proteins: Imp-*α*1,2 and 3 [31]–[34]. Based on the results of rescue experiments, the three Imp-α proteins are generally functionally redundant, although specialized roles in gametogenesis have been found for Imp-α1 and Imp-α2; however, neither is essential to survival [34]–[36]. On the other hand, Imp-α3 is required for larval survival and development of larval and adult structures [33].

In a similar fashion to NLSs, nuclear export signals (NESs) are recognized by specific exportin proteins which shuttle proteins though the NPC and into the cytoplasm. The best characterized exportin is Chromatin Region Maintenance 1 (Crm1), though a variety exportins and NESs exist (reviewed in [29], [30]). Crm1 recognizes hydrophobic NESs that are typically L/I rich, with a classical consensus of (LX{2,3}[LIVMF]X{2,3}LX[LI]), where X is any amino acid [37]. However, there are many examples of functional Crm1 dependent NESs that do not fit this pattern. For example, when this consensus was originally derived, a NES that was known to deviate from this pattern had been discovered in the equine infectious anemia virus Rev protein [38]. Recently, Kusugi *et al* tested a large set of artificially generated NESs for their ability to facilitate Crm1 mediated nuclear export and used these results to generate six classes of consensus sequences (1a–d, 2 and 3), which were then compared to experimentally derived signals ([39], and see the NES database at NESbase (http://www.cbs.dtu.dk/databases/NESbase/), [40]). *Drosophila* has a single ortholog of *Crm1* called *embargoed* (*emb*).

Herein, we show that Sd contains a *bona fide* bipartite classical NLS (cNLS), which is competent to direct an eGFP signal to the nucleus and is required for the proper nuclear targeting of Sd. We also identify a putative NES within Sd. Furthermore, we show that the C-terminal domain of Sd is also able to influence the localization of the protein, although the mechanism by which it does so is unknown. Lastly, we show that Sd which is targeted to the cytoplasm, or Sd which has a mutated NLS act in a dominant negative fashion and are unable to rescue wing development in a mutant background.

## Results

### Sd contains a putative NLS matching the classic bipartite sequence, which is conserved in many TEAD family members

**Figure 1:**
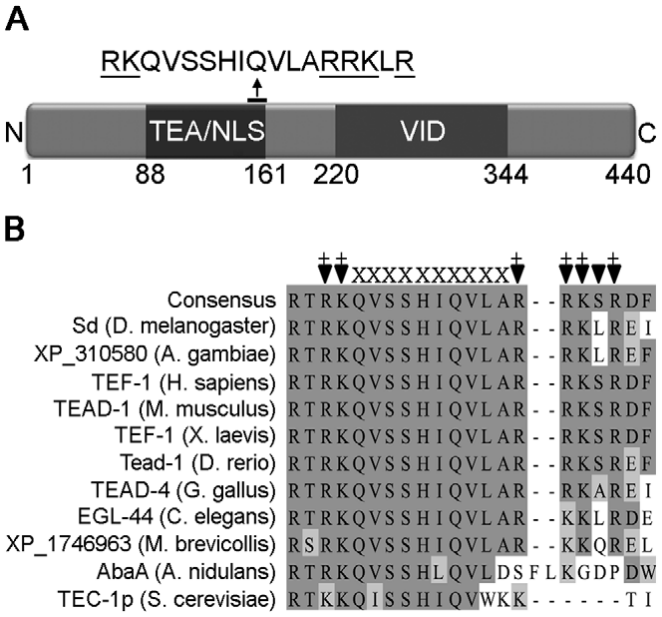
Identification of a putative bipartite NLS. (A) A schematic diagram of Sd. Sd contains two known functional domains, the TEA (DNA binding) domain and the Vestigial interacting domain (VID), as shown. At the C-terminus of the TEA domain, there is a 17 amino acid stretch from R145 to R161 which closely matches the consensus classic bipartite NLS sequence ([K/R]_2_[X]_10_[K/R]_>3/5_). (B) The region corresponding to the bipartite NLS shows strong identity with a variety of TEAD proteins from both animals and *Choanozoa* protists. Arrowheads mark the sites of the two N-terminal and five C-terminal residues known to be important for the classical bipartite sequence. ‘X’ marks the 10 intervening amino acids lying between the two termini. A ‘+’ indicates a basic residue (L/R) lies at one of the N- or C-terminal critical sites in the consensus sequence of the aligned TEAD proteins. The dark shading indicates identity with the consensus, while the lighter shading indicates similarity.

Using *in silico* analysis, an NLS fitting the consensus of the bipartite family of signals (see introduction) which could account for the theorized ability of Sd to translocate itself and its binding partners to the nucleus was previously identified [20], [41]. The sequence of this signal is RKQVSSHIQVLARRKLR, which is a close match to the classical consensus of [(R/K)_2_X_∼10_(R/K)_>3/5_] mentioned above (Fig. 1A; [41]). Moreover, the amino acids comprising this putative NLS are highly conserved among TEAD family members from species within both *Choanozoa* and *Animalia* (Fig. 1B). However, the C-terminal portion of the NLS is not conserved in the more distantly related Fungi.

### The NLS within Sd is sufficient to target an eGFP reporter to the nucleus

**Figure 2:**
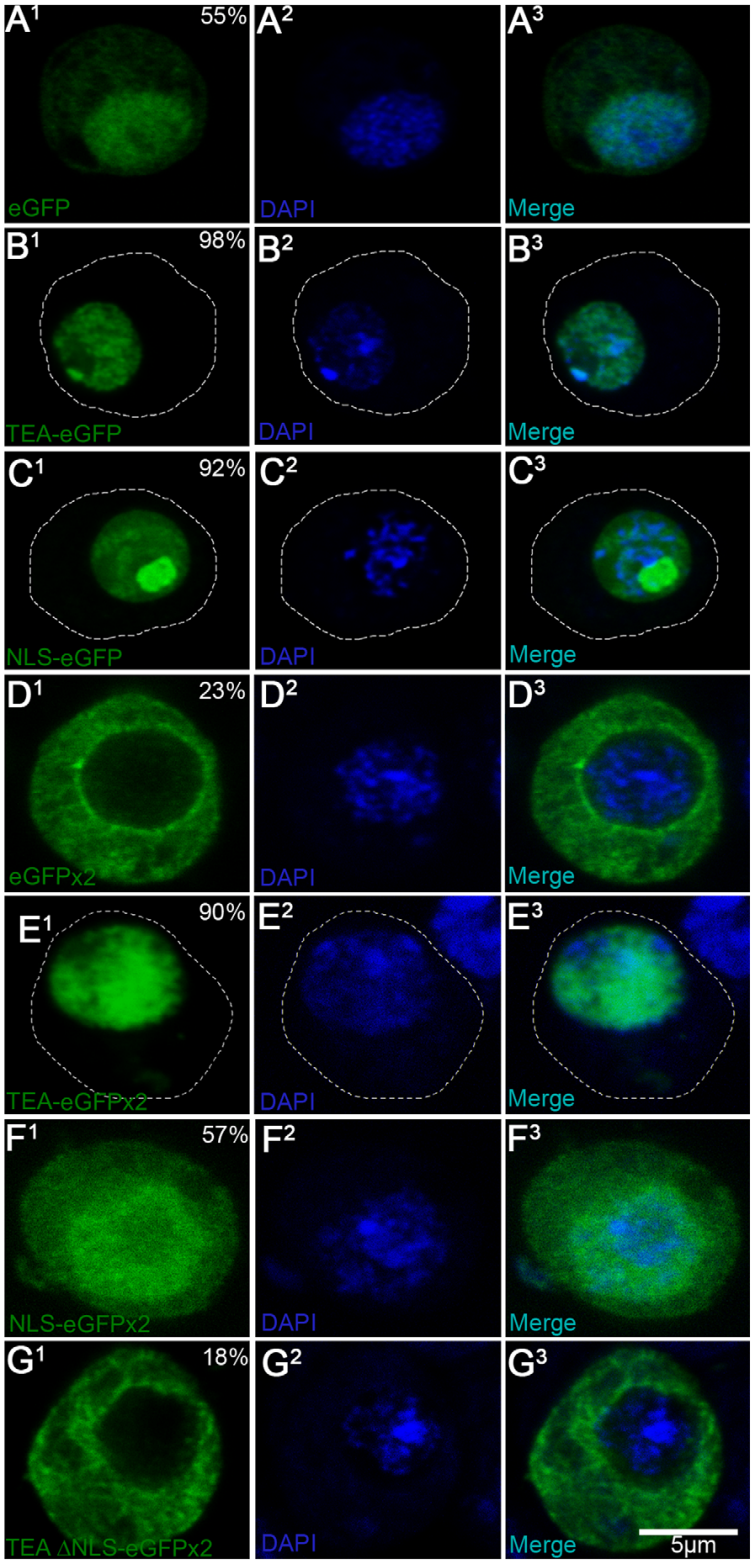
The NLS of Sd is directs an eGFP tag to the nucleus. (A–G) Localization of the indicated eGFP reporter tagged peptides in transiently transfected in S2 cells with DAPI stained nuclei and visualized via confocal microscopy. A^1^–G^1^ are the green (eGFP) channels. A^2^–G^2^ are the blue (DAPI) channels. A^3^–G^3^ are the green and blue channels (merge). Hatched lines indicate the boundary of cells, as determined by the extent of the weak cytoplasmic signal. Percentages indicate the percent nuclear signal relative to total signal measured in the given cell. (A) eGFP. When eGFP is expressed alone, diffuse expression is seen throughout the cell, including the nucleus. (B) TEA-eGFP. A fragment of Sd stretching from amino acids 88–178 (which includes the entire TEA/NLS domain) shows almost exclusive reporter activity within the nucleus of the expressing cells. (C) NLS-eGFP. Amino acids 143–163 of Sd (which includes the NLS and two flanking amino acids on either side) drives reporter expression to the nucleus. (D) eGFPx2+HA (referred to hereafter as eGFPx2). eGFPx2 expression is excluded from the nucleus. (E) TEA-eGFPx2. A TEA-eGFPx2 fusion is primarily nuclear. (F) eGFPx2+NLS. This construct is found throughout the cell, but is enriched in the nucleus. (G) TEAΔNLS-eGFPx2. When the NLS is removed from the TEA domain, it is no longer able to direct the tag to the nucleus.

In order to confirm the function of the putative NLS of Sd, we elected to tag the protein with an eGFP reporter and express the fusion proteins (under the control of a heat shock driver) in *Drosophila* S2 cells. The results of the experiments listed below are summarized in Table 1. When eGFP is expressed alone, diffuse signal is observed throughout the cytoplasm and nucleus of the cells, with ∼61% of the total signal located in the nuclei of cells, on average (Fig. 2A). This is likely because the small size of eGFP (∼27 kDa) enables it to pass through the NPC via passive diffusion. It has been previously shown that a chimeric protein consisting of amino acids 63–211 of Sd and full-length Vg is able to substitute for endogenous Sd function during wing development [20]. This, combined with the presence of the predicted bipartite sequence within this stretch of amino acids, implied that this region of Sd is sufficient to permit nuclear translocation of the complex. To verify this, we expressed a reporter construct containing a fragment of Sd which contained both the TEAD and the putative NLS signal (amino acids 88–174). In this case over 90% of the signal is nuclear in S2 cells (Fig. 2B). Extending this further, amino acids 143–163 (the predicted NLS extended by two amino acids on either side) were also sufficient to strongly target eGFP to the nucleus (88% nuclear; Fig. 2C). The large increase in nuclear signal compared to eGFP alone, suggests that these fusion peptides are being translocated much more efficiently. However, these two fusion peptides are both smaller than 40 kDa, so it is also possible that nuclear retention, rather than nuclear translocation, has been increased. To eliminate this possibility, we also tested the ability of the TEAD, the NLS and the TEAD lacking the NLS (amino acids 88–144 of Sd) to drive eGFPx2+glutathione S-Transferase (hereafter referred to as simply eGFPx2) to the nucleus. Unlike eGFP alone, this tag is very large (94 KDa) and is almost completely excluded from the nucleus (Fig. 2D and see [42]. As before, both the TEAD and NLS of Sd are able to shift the localization of this tag to the nucleus (Figs. 2E and F), giving 79% and 60% nuclear signal, respectively. Conversely, the TEAD lacking the NLS failed to drive the protein tag to the nucleus, as less than 20% of the observed signal was nuclear (Fig. 2G). As a general observation, we noted that eGFP and NLS-eGFP appeared to be able to localize to the nucleolus, while all other constructs tested (including those described below) were largely excluded from this region.

**Table 1:** Quantification of the cellular distribution of the eGFP tagged peptides.

Construct (KDa)	N	Average %Nuc./Total (S.E.M.)	%Nuc. (>80%)	%Diffuse Nuc. (79-58%)	%Diffuse (57-36%)	%Excl. (<35%)
eGFP (29.7)	20	61.1 (0.9)	0.0	80.0	20.0	0.0
TEA-eGFP (39.6)	25	94.6 (0.9)	100.0	0.0	0.0	0.0
NLS-eGFP (32)	34	88.2 (1.2)	85.3	14.7	0.0	0.0
eGFP-Sd (78.7)	32	92.5 (0.6)	100.0	0.0	0.0	0.0
eGFPx2 (82.9)†	25	22.5 (1.2)	0.0	0.0	8.0	92.0
TEA-eGFPx2 (94)	19	78.8 (2.4)	57.9	42.1	0.0	0.0
TEAΔNLS-eGFPx2 (91.9)†	19	14.8 (0.7)	0.0	0.0	0.0	100.0
eGFP-SDΔNLS (76.6)†	31	42.6 (1.1)	0.0	3.2	83.9	12.9
eGFP-SD mNLS^N^ (78.5)	37	79.6 (1.6)	51.4	43.2	5.4	0.0
eGFP-SD mNLS^C^ (78.5)†	38	46.9 (0.8)	0.0	0.0	100.0	0.0
eGFP-SD mNLS^N+C^ (78.5)†	35	44.1 (1.3)	0.0	2.9	91.4	5.7
NES-eGFP (32.5)†	44	45.1 (2.1)	0.0	22.7	52.3	25.0

**Figure 3:**
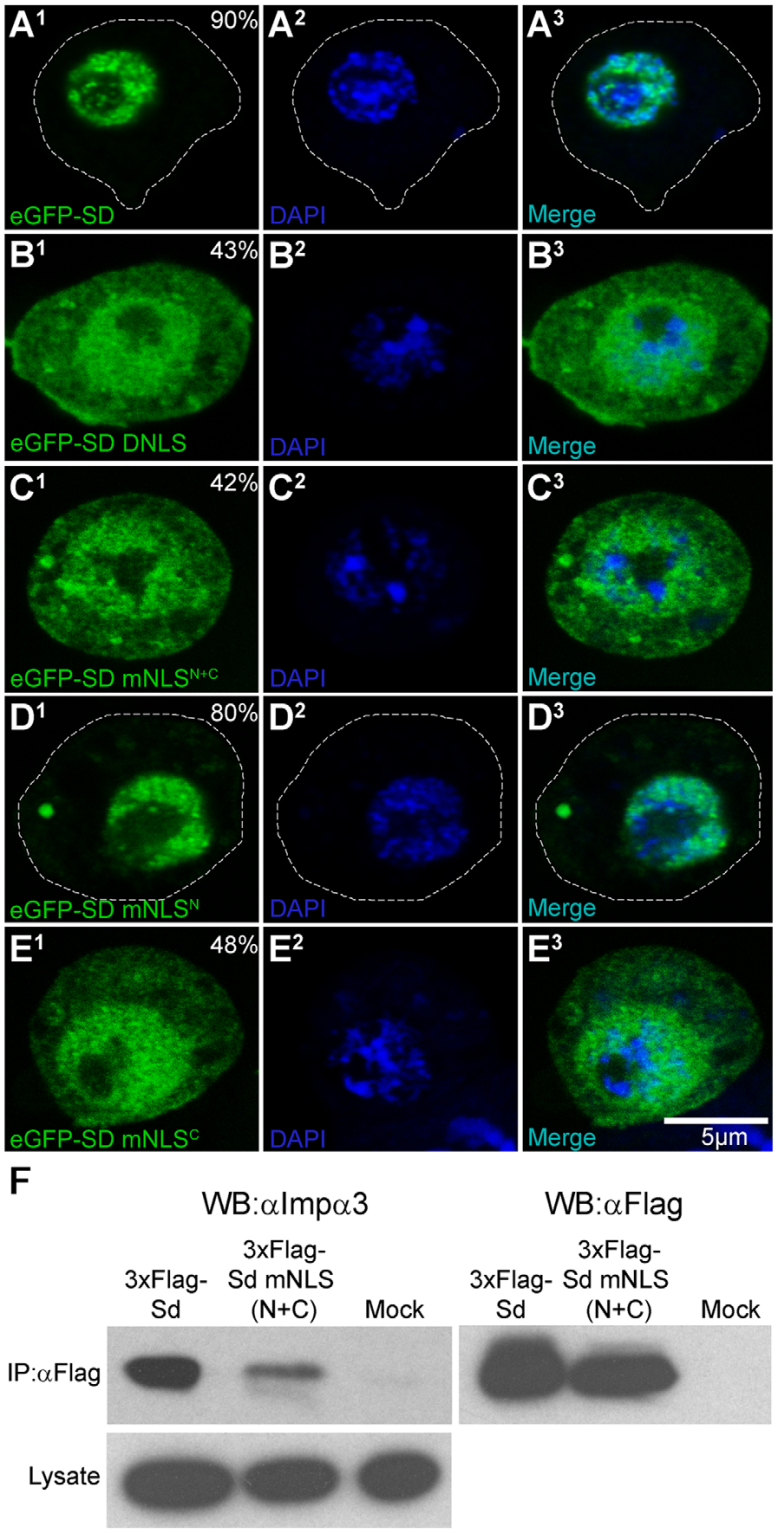
The intact NLS is necessary for both the proper nuclear translocation of Sd and Importin-α3 binding. (A–E) Localization of the indicated eGFP reporter tagged proteins in transiently transfected in S2 cells with DAPI stained nuclei and visualized via confocal microscopy. See the legend for Figs. 2A–G for details. (A) eGFP-SD. When Sd is expressed in S2 cells, reporter activity is predominantly nuclear. (B) eGFP-SD ΔNLS. Deleting amino acids 143–163 of Sd disrupts its localization and leads to diffuse reporter activity throughout both the nucleus and cytoplasm. (C) eGFP-SD mNLS^N+C^. Mutation of the six basic amino acids identified as being part of the classical consensus bipartite sequence (see Fig. 1) to N causes disruption of localization similar to that seen when the NLS is deleted. (D) eGFP-SD mNLS^N^. When the two N-terminal basic amino acids are mutated to N, a lesser disruption of the nuclear signal is observed (compare to A). (E) eGFP-SD mNLS^C^. Sd with the four C-terminal basic amino acids mutated to N drives diffuse localization of the eGFP reporter, similar to that seen for SD ΔNLS and SD mNLS^N+C^. (compare to panels B and C, respectively). (F) Co-IP of Sd and Imp-α3. Cells expressing 3xFLAG-Sd, 3xFLAG-Sd mNLS^N+C^ as well as cells mock transfected with water alone were lysed, immunoprecipitated with αFLAG beads and analyzed via western blotting. Detection was with anti-FLAG or anti-Imp-α3. Detection with anti-FLAG ensures expression of the two tagged proteins is approximately equal. The lysate of all cells had a strong Imp-α3 signal. Imp-α3 co-immunoprecipitated strongly with 3xFLAG-Sd, while only weakly with 3xFLAG-Sd mNLS^N+C^. The mock transfected cells showed almost no Imp-α3 signal after immunoprecipitation, controlling for the specificity of the anti-FLAG beads.

The eGFP fusion constructs from Figs. 2A–G, 3A–E and Fig. 5D were assayed for the percentage of eGFP signal seen in the nuclei of the expressing cells; see materials and methods. (S.E.M) is the standard error of the mean. A † denotes a construct with diffuse or nuclear excluded signal (<58% nuclear signal). N is the total number of cells measured from at least two independent transfections. The next four columns represent four arbitrary localization patterns along with the mean nuclear signal each grouping represents. For each peptide, the percentage of cells that fall into one of the four categories is indicated. The means of the experimental constructs TEA-eGFP, NLS-eGFP and NES-eGFP are statistically different from their control (eGFP) at p<0.001. Likewise, NLS-eGFPx2, TEA-eGFPx2 and TEAΔNLS-eGFPx2 are significantly different from eGFPx2, at p<0.0001. Finally the mean of the control eGFP-Sd was significantly different from the four reporter constructs in which the NLS was mutated, at p<0.001. Nuc. = Nuclear. Excl. = Excluded.

### The NLS is necessary for the proper nuclear localization of Sd as well as efficient Importin-α3 binding

When expressed in S2 cells, eGFP-Sd shows very strong nuclear localization (Fig. 3A). When the NLS was either deleted (Sd ΔNLS, Fig. 3B) or the six basic amino acids (R145, K146, R157, R158, K159 and R161) identified in Fig. 1A were mutated to asparagines (Sd mNLS^N+C^; Fig. 3C), the ratio of nuclear signal to total signal is reduced to less than 50%, compared to greater than 90% for intact Sd. This provides evidence that the identified NLS is required for the proper ex vivo localization of Sd. See Table 1 for a summary of these results and those that follow.

Extending this analysis, tagged Sd isoforms were generated where only the N-terminal basic amino acids (R145 and K146), or the C-terminal basic amino acids (R157, R158, K159 and R161) are mutated to asparagines (Sd mNLS^N^ and Sd mNLS^C^, respectively). When the N-terminal amino acids are mutated, a small but significant (p<0.001) increase in cytoplasmic signal is observed (Fig. 3D) and the nuclear fraction is reduced to ∼80%. Conversely, mutating the C-terminal basic amino acids results in diffuse localization of the eGFP signal to both the nucleus and cytoplasm (Fig. 3E). The magnitude of mis-localization is similar to that seen when the entire NLS is deleted or both clusters of basic amino acids are mutated, with less than 50% of the total signal seen in the nucleus. Surprisingly, regardless of which method of NLS disruption was employed, a significant fraction (>40%) of signal was still observed in the nucleus of expressing cells.

As mentioned previously, Imp-α3 appears to be generally required throughout development and so we elected to test both the ability of this protein to bind Sd, and whether this binding was dependent on the NLS of Sd. To do this 3xFLAG-tagged Sd or Sd mNLS^N+C^ were expressed in S2 cells and tested for the ability to co-immunoprecipitate (Co-IP) endogenous Imp-α3. A mock transfection was also done using water alone. While Imp-α3 was detected in the lysate of all three types of transfected cells, only 3xFLAG-Sd and, to a much lesser extent, 3xFLAG-Sd mNLS^N+C^ were able to Co-IP Imp-α3 (Fig. 3F).

### Discrete regions within the C-terminal domain of Sd act to facilitate or repress nuclear localization

**Figure 4:**
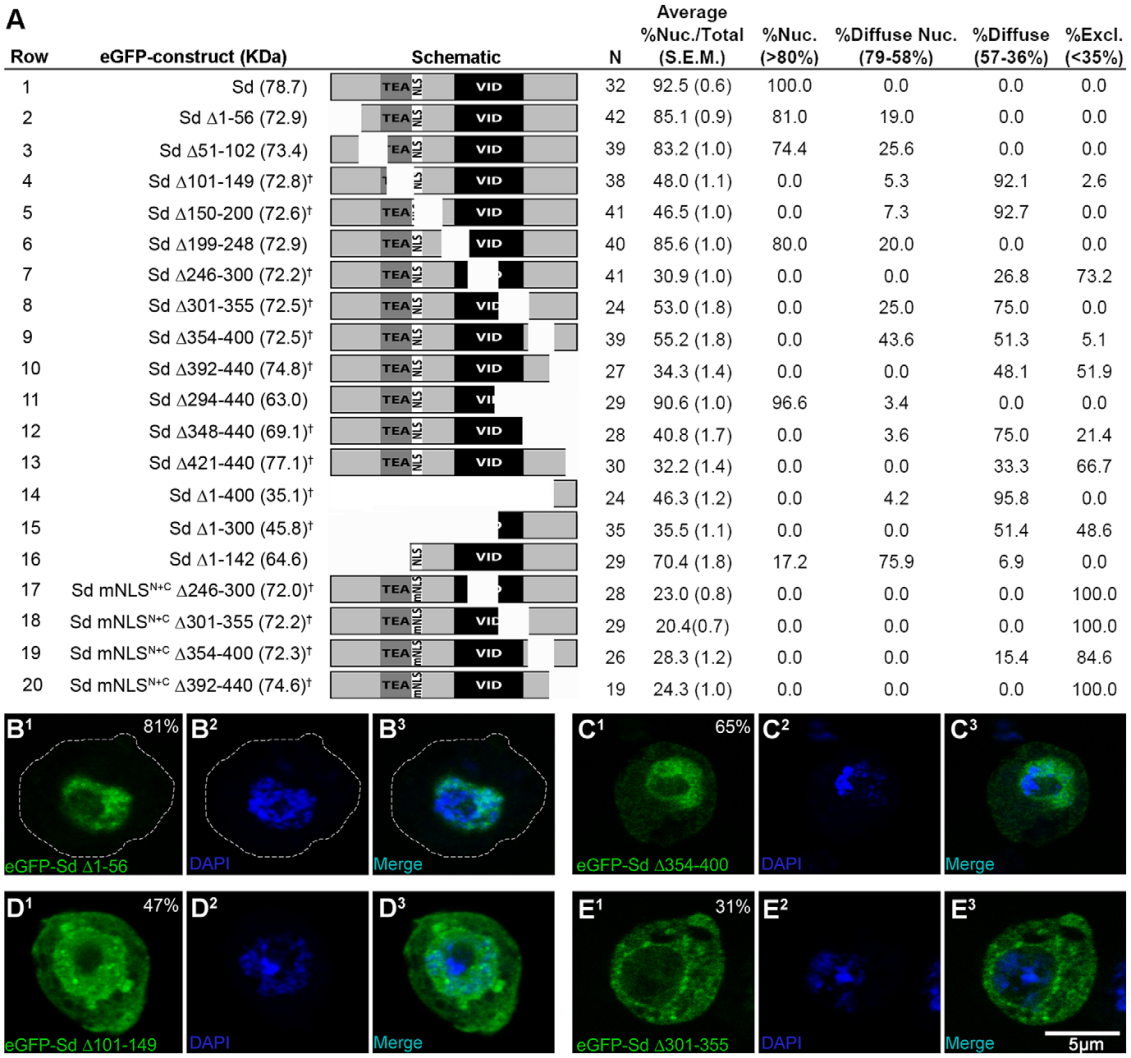
The C-terminal domain can act to both repress and facilitate the nuclear localization of Sd. A series of internal deletions and truncations of Sd were generated, expressed with a fused N-terminal eGFP marker in S2 cells and assayed for cellular distribution. (A) Schematic of the various Sd isoforms generated along with a summary table of the localization experiments. The domains of Sd are as described in Fig. 1A. ‘mNLS^N+C^’ is described in Fig. 3C. A † denotes a construct with diffuse or nuclear excluded signal (<58% nuclear signal). N is the total number of cells measured from at least two independent transfections. The next four columns represent four arbitrary localization patterns along with the mean nuclear signal each grouping represents. For each peptide, the percentage of cells that fall into one of the four categories is indicated. (B–E) Representative cells showing 81%, 65%, 47% and 31% nuclear signal (B, C, D and E, respectively). See the legend for Figs. 2A–G for details.

There are many examples of proteins which contain multiple signals/regions which influence (in both a positive and negative fashion) the localization of the protein (for examples see [43]–[46]). Given our results, it was hypothesized this might be true for Sd as well. To test this, a complete series of ∼50 aa deletions of Sd were generated and assayed for the ability to drive eGFP to the nucleus (Fig. 4A). Three deletions (Sd Δ1–56, Sd Δ51–102 and Sd Δ199–248) which in all cases leave the NLS intact, showed a small decrease in the ratio of nuclear to cytoplasmic signal of ∼7–9%, relative to full-length Sd (Fig. 4A, rows 2,3 and 6 compared to row 1). As the deletions are significant in size, this minor perturbation is likely due to overall changes to the tertiary structure of the deletion molecules, rather than the disruption of specific signals. A fourth construct, deleting the N-terminus portion of Sd up to the NLS was also tested (Sd Δ1–142, Fig. 4A row 16). In this case the localization was reduced further relative to the other N-terminal deletions (70.4% nuclear vs. 85.1% and 83.2% for Sd Δ1–56 and Sd Δ51–102, respectively). However, this reduction of ∼24% relative to wildtype is still less severe than those seen in deletions encompassing the NLS or the C-terminal domain of Sd (see below). Additionally, disrupting both the NLS and C-terminal domain, but leaving the TEAD otherwise intact, essentially abolishes all signal in the nucleus (Fig. 4A, rows 17–20 and see below).

The five other deletions (Sd Δ101–149, Sd Δ246–300, Sd Δ301–355, Sd Δ354–400 and Sd Δ392–440) all had a greatly reduced nuclear signal relative to cytoplasmic signal, as compared to full-length Sd (ranging from a 40% reduction with Sd Δ354–400 to a 67% reduction with Sd Δ246–300; Fig. 4A rows 4,5 and 7–10). The first, Sd Δ101–149, disrupts the NLS of Sd, lending further support to the notion that this domain is required for Sd localization. The other four deletions either disrupt the Vestigial interacting domain, (VID, Sd Δ246–300 and Sd Δ301–355) or the remainder of the C-terminal domain of Sd (Sd Δ354–400 and Sd Δ392–440). A small 20 amino acid deletion at the C-terminus of Sd is also able to reduce the ratio of nuclear signal to total signal by 65%, relative to full length Sd (Sd Δ421–440, Fig. 4A row 13). These data show that large portions of the C-terminal domain of Sd, including the VID, are necessary for Sd to direct the eGFP tag to the nucleus of S2 cells. However, this domain cannot direct eGFP to the nucleus alone since both Sd Δ348–440 and Sd Δ1–400 are located predominantly in the cytoplasm. Interestingly, mutating the seven critical basic amino acids of the NLS in conjunction with each of the four large deletions in the C-terminus (Sd mNLS^N+C^ Δ246–300, Sd mNLS^N+C^ Δ301–355, Sd mNLS^N+C^ Δ354–400 and Sd mNLS^N+C^ Δ392–440) results in a phenotype considerably stronger than that when only the NLS is mutated or only the deletions are present. Indeed, three of these constructs were exclusively cytoplasmic in all cells studied, while the fourth, Sd mNLS^N+C^ Δ354–400, was exclusively cytoplasmic >80% of the time and showed a diffuse localization in the remainder of the cells examined (Fig. 4A rows 17–20 and compare to Fig. 3C and Table 1). Additionally, two known alleles of *sd*, *sd^68L^* and *sd^11L^* previously mapped to the C-terminal coding region of *sd* [27] were generated as eGFP fusion constructs and expressed in S2 cells. The mutant fusion proteins generated both localized strongly to the nucleus (data not shown).

Contrary to the deletion results detailed above, Sd molecules truncated just downstream of the beginning of the VID or roughly half-way into the VID (Sd Δ229–440 and Sd Δ294–440) locate strongly to the nucleus (>90% nuclear signal), even though they lack the more C-terminal portions of the molecule shown to be important via the previously described deletion analysis (data not shown and Fig. 4A row 11, respectively). An additional series of truncations was generated to further narrow down potential signals in this last region. As mentioned above, Sd Δ348–440 showed a mis-localization phenotype, with less than 41% of the signal being nuclear (Fig. 4A row 12). Truncations further C-terminal to amino acid 347 (Sd Δ374–440 and Sd Δ401–440) also had a strong mis-localization phenotype (data not shown). These results imply that amino acids 294–348 interfere with nuclear localization in some fashion, at least in the absence of the remainder of the C-terminus. Consistent with these results, a construct containing the majority of these amino acids (Sd Δ1–300) shows strong cytoplasmic signal with only 35.5% nuclear signal on average and almost half of the cells showing nuclear exclusion of the eGFP signal (Fig. 4A row 15). However, it should be mentioned that the previously mentioned internal deletion Sd Δ301–355, is largely cytoplasmic, yet also deletes the majority of this region. Representative cells for the described phenotypes are shown as Figs. 4B–E.

One potential flaw in the previous analysis is that the deletions generated may have an impact on protein structure and/or stability and therefore the changes in localization seen may be a secondary effect of the deletions, rather than a primary effect due to the removal of targeting signals. While it is impossible to rule out this possibility completely, there are a few lines of evidence to counter this line of reasoning: First, a few deletions (Sd Δ301–355 and Sd Δ392–440) were tested with a C-terminal GFP tag, rather than an N terminal tag. No significant difference in localization between the C-tagged forms and the N-tagged form were seen (data not shown). Secondly, unstable proteins which are abundantly expressed would be expected to form aggregates known as inclusion bodies (reviewed in [47]). While a small amount of aggregation is seen, the relative levels appear to be low especially given that eGFP alone is known to aggregate readily, thus is seems unlikely that the results above are only due to protein instability.

### The region antagonizing Sd nuclear localization contains a putative NES and is responsive to Leptomycin B

**Figure 5:**
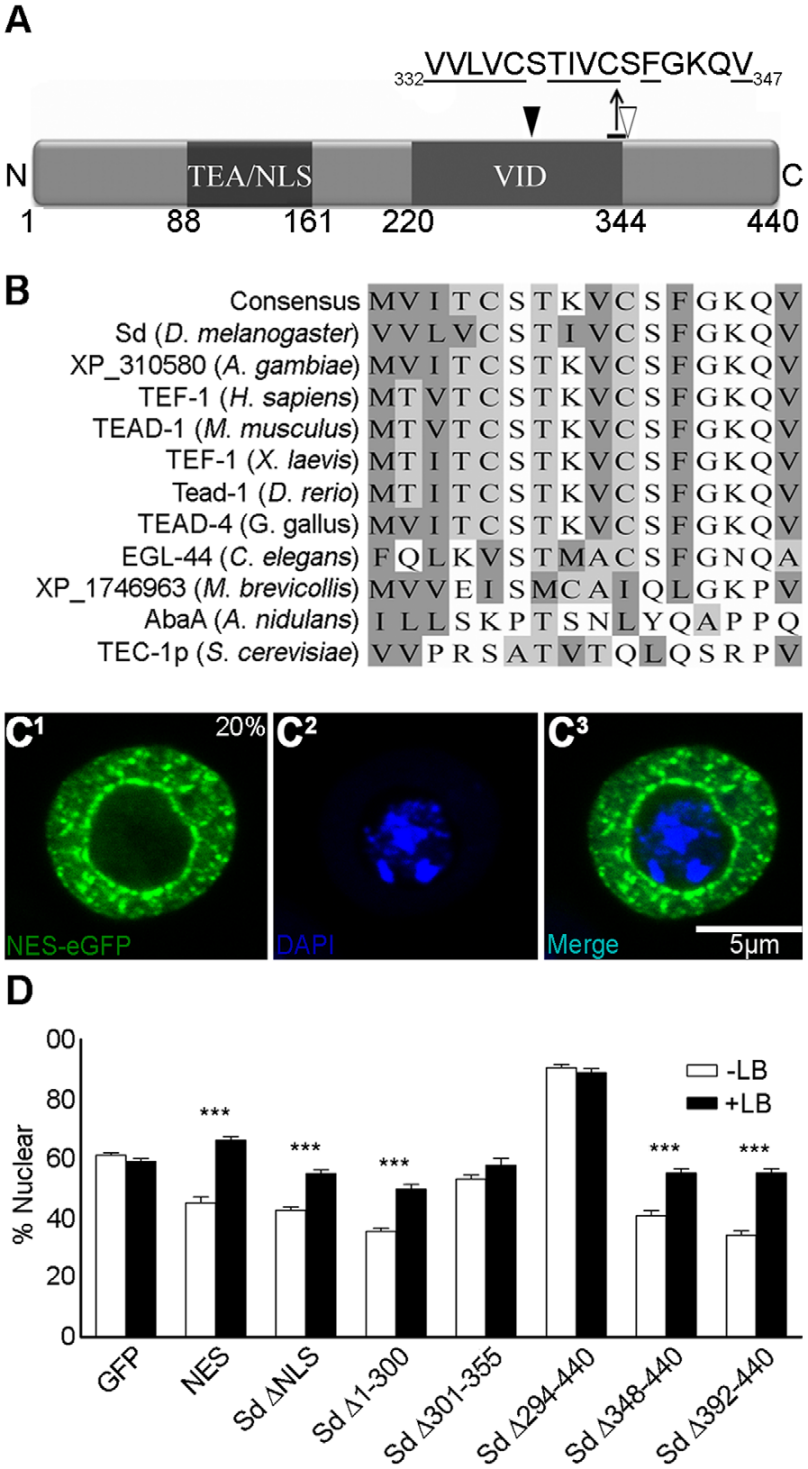
Sd contains a sequence at amino acids 332–347 which increases the cytoplasmic fraction of a fused eGFP tag in a leptomycin B (LB) sensitive manner. (A) Schematic of Sd with the putative NES marked. The domains of Sd are described in Fig. 1A. Hydrophobic residues are underlined. The open and closed arrowheads mark the boundaries of the region intact in SD Δ344–440 and missing in SD Δ294–440, respectively. (B) Alignment of several TEAD proteins. Dark shading indicates hydrophobic residues L, I, V, M and F, while light shading indicates hydrophobic residues C, W, A or T. (C) Sd amino acids 330–347 were fused N-terminal to eGFP and assayed for spatial distribution. A representative cell showing nuclear exclusion of the fusion protein is shown. See the legend for Figs. 2A–G for details. (D) Nuclear fraction of eGFP tagged constructs in LB treated and untreated S2 cells. eGFP-tagged isoforms of Sd which contained the NES (NES, Sd ΔNLS, Sd Δ348–440 and Sd Δ392–440), and Sd fragments in which the NES was deleted (Sd Δ301–355 and Sd Δ294–440), were tested for nuclear localization in the presence and absence of Leptomycin B. N is >15 for all conditions. *** indicates a significant difference at P<0.001. Error bars are the standard error of the mean.

Based on the results described in the previous section, amino acids 294–347 of Sd act to inhibit nuclear localization in some fashion. Within this stretch of amino acids, there is a region with an abundance of hydrophobic residues (11/16 residues, not including K), beginning at V332 and ending at V347 (Fig. 5A). Although the identity of the residues differs slightly between family members, this hydrophobic region is also present in TEAD proteins from *Choanozoa* and *Animalia* (Fig. 5B). The consensus of this region in these proteins contains hydrophobic residues in 10/16 positions total, and these residues align with those in Sd with the exception of residue I339. This residue is hydrophobic in only 4/11 of the species examined (Fig. 5B). The hydrophobic region of Sd can be aligned with four of the NES classes (1a, 1b, 1d and 3), while the consensus sequence aligns with three of the NES classes (1a, 1b and 3) described by Kusugi *et al* (Table 2; [48]).

**Table 2:** Alignment of TEAD proteins with four classes of NESs.

NES Class	Consensus Sequence	Sd Sequence	Equivalent TEAD Consensus Sequence
1a	Φ-3X-Φ-2X-Φ-X-Φ	**V** VLV**C**ST**I**VCS**F**GKQV	N/A
		VV**L**VCS**T** IV**C**S**F**GKQV	MV**I**TCS**T**KV**C**S**F**GKQV
1b	Φ-2X-Φ-2X-Φ-X-Φ	**V** VL**V**CS**T** I**V**CSFGKQV	**M** VI**T**CS**T**K**V** CSFGKQV
		V**V**LV**C**ST**I**V**C**SFGKQV	N/A
		VVL**V**CS**T** IV**C**S**F**GKQV	MVI**T**CS**T**KV**C**S**F**GKQV
1d	Φ-2X-Φ-3X-Φ-X-Φ	**V** VL**V**CST**I**V**C**SFGKQV	N/A
2	Φ-X-Φ-2X-Φ-X-Φ	V**V**L**V**CS**T** I**V**CSFGKQV	M**V**I**T**CS**T**K**V** CSFGKQV
		VV**L**V**C**ST**I**V**C**SFGKQV	N/A

Four classes of NESs derived from a comparison to natural and synthetic NESs are indicated. In the third column, the Sd hydrophobic region is aligned to fit these patterns, and if possible, the fourth column shows the equivalent residues from the TEAD protein consensus. Within a consensus sequence, an underline represents a hydrophobic residue. A bolded and enlarged hydrophobic residue is one that is compatible with the associated NES class pattern.

To test the possibility that this region contains an NES, a small peptide which includes the putative NES region (Q325 to E352) was fused N-terminally to eGFP (NES-eGFP) and expressed in S2 cells. This caused the average nuclear fraction to be reduced by ∼26%, relative to eGFP alone. Moreover, contrary to eGFP, which never showed nuclear exclusion, the NES-eGFP expressing cells examined showed nuclear exclusion of the eGFP tag (Fig. 5C) 25% of the time. The other distributions seen were also quantified and tabulated in Table 1. Compared to eGFP which showed an enrichment of nuclear signal 80.0% of the time, this distribution was observed in only 22.7% of the NES-eGFP expressing cells. Finally, 55.3% of NES-eGFP cells showed more diffuse localization, compared to 20.0% for eGFP alone. Altogether, although NES-eGFP had a range of phenotypes, some of which overlapped eGFP, the presence of the hydrophobic region of Sd generally decreased the amount of nuclear signal observed and resulted in nuclear exclusion in many cases.

Leptomycin B (LB) is a potent inhibitor of Crm1 dependent nuclear export [49], [50]. Thus, we tested the ability of this chemical to influence the sub-cellular trafficking of NES containing constructs (Fig. 5D). When LB is added to cells expressing eGFP alone, no significant change in localization is seen. Similarly, Sd Δ301–355 and Sd Δ294–440 (which lack the NES described above) do not show a response to LB treatment. On the other hand, the NES-eGFP construct is responsive to LB, as are deletion constructs which are lacking the NLS but contain the NES (Sd ΔNLS and Sd Δ1–300). Furthermore Sd isoforms which contain both the NLS and NES, but are disrupted more C-terminally to the NES (Sd Δ348–440 and Sd Δ392–440) are also rescued by the addition of LB.

### 3xFLAG-PMSD and SD mNLS^N+C^ are potent dominant-negative forms of *sd* and cannot substitute for wild-type Sd in wing development

**Figure 6:**
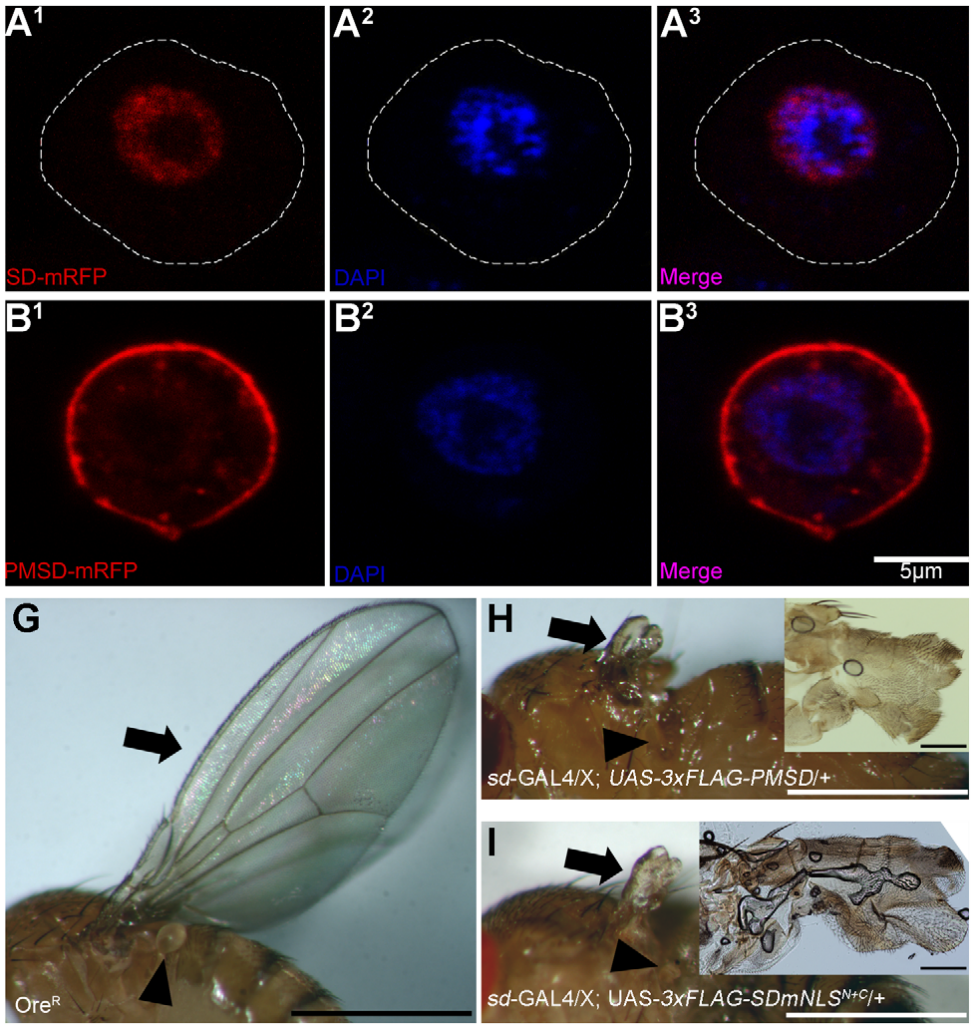
During wing development, 3xFLAG-PMSD and 3xFLAG-SD mNLS^N+C^ act as dominant negative forms of Sd. (A and B) Localization of the indicated mRFP reporter tagged proteins in transiently transfected in S2 cells with DAPI stained nuclei and visualized via confocal microscopy. See Figs. 2A–G for details. (A) Sd-mRFP expression. Sd strongly localizes an mRFP tag to the nucleus. (B) PMSD-mRFP expression. Sd tagged with a N-terminal palmitoylation/myristoylation sequence (PMSD) and C-terminal mRFP tag shows strong localization to the cytoplasmic membrane of S2 cells. (C–E) Light micrographs of flies with the indicated genotypes. (C) Wildtype Oregon-R (Ore^R^) fly. (D–E) Males containing either *UAS*-*3xFLAG*-*PMSD* or *UAS*-*3xFLAG*-*SD mNLS^N+C^* (see Fig. 3C) inserted on the 2^nd^ chromosome and balanced over *CyO* were crossed (two independent lines/insert) to virgin females homozygous for *sd*-GAL4 and the resultant progeny were scored. Insets are magnified views of the wing tissue. Scale bars are 1 mm (D–E) or 0.1 mm (D and E insets). Arrows indicate the wing, while arrowheads indicate the haltere. (D) Female fly containing *UAS*-*3xFLAG-PMSD* under the control of *sd*-GAL4. Almost no wing or haltere tissue is present. (E) Female fly containing *UAS*-*3xFLAG-mNLS^N+C^* under the control of *sd*-GAL4. Again, virtually no wing or haltere tissue is present.

To test for the necessity of Sd nuclear localization *in vivo*, we constructed a Sd protein that contains a Yes palmitoylation/myristoylation (pal/myr) signal as well as a Fyn linker sequence appended to the N-terminal domain of Sd (PMSD). This sequence is known to target eGFP to the plasma membrane and endosomes [51]. Indeed, fusing this sequence to Sd and a monomeric red fluorescent protein (mRFP) tag likewise targets this fusion protein to these same locations (Fig. 6B), rather than the nucleus as is the case for Sd lacking the (pal/myr) signal (Fig. 6A). Two transgenic lines (3-2 and 4-1) each containing a flag-tagged form of this construct (UAS-*3xFLAG-PMSD*) were generated, and the transgene was expressed under the control of a *sd*-GAL4 driver. In these crosses, 76 and 111 progeny were scored, respectively. The majority of the progeny of the first cross were females (45%) or males (34%) which inherited a balancer chromosome, rather than the transgene. The remaining 21% of the flies were females with greatly reduced wings and halteres (Figs. 6D), relative to a Oregon-R (Ore^R^) fly (Fig. 6C). No non-balancer male progeny were observed. In the second cross, 29% and 21% of the progeny were females or males, respectively, which inherited the balancer chromosome. Furthermore, 27% of the progeny were females with greatly reduced wings and halteres similar to those seen when the 3-2 line was used. Contrary to the 3-2 line, the 4-1 line also yielded male progeny with this phenotype. These flies accounted for 23% of the total progeny. Transgenic flies containing a flag-tagged UAS-*3xFLAG-SD mNLS^N+C^* transgene were also generated. A similar range of progeny phenotypes was also seen when a UAS-*3xFLAG-SD mNLS^N+C^* was expressed using the *sd* driver. Again two lines were used, A (39 progeny of the *sd*-GAL4 cross scored) and B (62 progeny of the *sd*-GAL4 cross scored). When line A was used, the distribution of progeny females with the balancer, progeny males with the balancer and progeny females with reduced wing/haltere tissue (Fig. 6E) was 50%, 42% and 8%, respectively. No non-balancer male flies were observed. The equivalent distribution observed when using line B was 45%, 19% and 32%. In this case males with the wing/haltere phenotype were seen 3% of the time. None of the progeny from any of the four crosses had any obvious defects outside those observed in the wing and haltere.

Over-expression of wildtype Sd is able to cause strong wing phenotypes in an otherwise wildtype background. However, in *sd* mutants which have a strong wing phenotype (*sd^58d^*; [1]) the same construct is also able to significantly restore wing development when driven with *vg*-GAL4 [19]. While both UAS-*3xFLAG-PMSD* and UAS*-3xFLAG-SD mNLS^N+C^* have a strong dominant negative effect in wildtype flies, as shown above, neither is able to rescue the wings of *sd^58d^* flies when driven with *vg*-GAL4 (data not shown).

The SV40 large T-antigen NLS is the prototypical cNLS and is known to be able to direct eGFP to the nucleus [52]–[54]. As such, we tested to see if this NLS was able to rescue our Sd NLS mutants by generating transgenic lines which contained a 3xFLAG-SV40NLS-Sd mNLS^N+C^ transgene. While the addition of this signal was able to increase the amount of eGFP-Sd mNLS^N+C^ found in the nucleus of S2 cells from ∼44% to 68%, no change in the *in vivo* dominant negative phenotypes were seen, and this isoform of Sd was still unable to rescue *sd^58d^* mutants (data not shown).

## Discussion

The data presented show the previously only predicted NLS of Sd is indeed functional. Both eGFP and eGFPx2-GST are targeted to the nucleus by the NLS of Sd, even though the latter is too big to undergo passive diffusion into the nucleus. Based on the sequence of the NLS, and the fact that this sequence facilitates Imp-α3 binding, this signal is likely a member of the bipartite family of cNLSs. Moreover, although mutating the N-terminal basic amino acids in the signal only has a minor effect on the strength of the signal, this is consistent with typical bipartite signals, where the N-terminal cluster of basic amino acids is less critical then the C-terminal cluster [48]. To our knowledge, this is the first such signal that has been confirmed to be functional within a TEAD containing protein. However, the signal is well-conserved and it is plausible that it is also functional in other representatives of this widespread and important family of transcription factors.

As mentioned, the NLS of Sd shows homology to the classically defined bipartite family. However, the sequence is not consistent with a more refined consensus derived by Kasugi *et al* [48]. These researchers compared published NLS sequences to randomly generated artificial sequences which were assayed for their ability to direct eGFP to the nuclei of various cell lines. In this way they generated two consensus sequences: KRX_10–12_K(K/R)X(K/R) and KRX_10–12_K(K/R)(K/R). Even though the NLS of Sd (RKQVSSHIQVLARRKLR) is similar to both of these patterns, it is unique in that RK, rather than KR, is found at the N-terminal portion of the signal and furthermore R, rather than K, is found at the first position of the C-terminus. Thus, the NLS of Sd is a novel member of the bipartite family of cNLSs.

It has been previously speculated that mutant forms of Sd, which retain the ability to interact with Vg and other co-factors but lack the ability to enter the nucleus or bind DNA, act in a dominant negative fashion by titrating the binding partners of Sd This in turn reduces the amount of these co-factors available to interact with endogenous Sd [10], [18], [19]. We have reinforced this idea by expressing isoforms of Sd which are either targeted to the cytoplasmic membrane and endosomes (3xFLAG-PMSD) or have a mutated NLS (3xFLAG-Sd mNLS^N+C^). Both these isoforms act as strong dominant negative forms of Sd during wing development, implying they are still able to interact and titrate endogenous Vg. However, neither is able to substitute for endogenous Sd in a *sd^58d^* mutant background, demonstrating that a critical function is impaired in both isoforms of Sd. In the case of 3xFLAG-PMSD, the protein has not been altered in any way, thus it is unlikely that anything other than the protein's sub-cellular localization has changed. By extension, the fact that 3xFLAG-Sd mNLS^N+C^ gives identical phenotypes to 3xFLAG-PMSD and that the NLS is clearly functional in S2 cells strongly suggests that localization is similarly impaired *in vivo*. Contrary to this, the SV40 NLS is not able to rescue the function of Sd mNLS^N+C^ *in vivo*, even though it can rescue localization *in vitro*. We do not believe these results are incompatible for two reasons: First, the magnitude of rescue in S2 cells was significant, but not complete. Therefore, it is possible that no effect is seen phenotypically. Second, the mutations fall within the DNA binding domain of Sd, and thus might have secondary effects on the protein's ability to function *in vivo*.

In addition to identifying a bipartite cNLS in Sd, we also identified putative NES which, if indeed functional, likely relies on Crm1 to facilitate nuclear export. Together with the presence of the NLS we identified, this raises the possibility that there is a switch between nuclear and cytoplasmic forms of Sd and that the protein may be capable of shuttling between the two domains under certain conditions. There is some evidence to support this idea; in mouse NIH3T3 cell culture, the TEAD protein Tead1 shows reduced nuclear localization in the presence of activated Hpo pathway components [55]. Furthermore, our data indicates that the domain C-terminal to the NES (amino acids 353–440) must have at least one other signal which facilitates nuclear import. The easiest explanation for this observation would be that Sd contains an additional NLS. Indeed, there have been other proteins discovered which rely on the presence of two or more NLS sequences for their proper nuclear import. For instance, the mammalian MSH6 protein (which is involved in DNA mis-match repair) contains three NLSs and it is only when all three are intact that MSH6 shows its proper nuclear localization [56]. That said, i*n silico* analysis did not identify any other regions which resemble an NLS, and the C-terminal domain of Sd is insufficient to target an eGFP tag to the nucleus. Thus it is unlikely that another NLS exists within this domain of Sd. Rather, all available evidence suggests that this domain is responsible for protein-protein interactions, since two (Yki and Vg) of the three known cofactors of Sd are known to bind to this domain [10], [14]. We favor the hypothesis that this domain allows Sd to bind a co-factor which, in addition to the NLS of Sd, facilitates the translocation of the complex to the nucleus. It is quite possible that one of the other proteins is endogenous Sd itself, since Sd is known to dimerize and there is evidence that Sd transcripts are present in S2 cells ([57], and our unpublished data). However, in the study by Ota and Sasaki mentioned previously, they showed that the Yki homologue Yap65 responds to Hpo signaling in a similar fashion as Sd – that is, it shows a reduction in nuclear localization. They also demonstrated that a mutant form of Yap65, lacking a target phosphorylation site, maintained strong nuclear localization in the presence of Hpo signaling. Moreover, this mutant form of Yap65 was also able to increase the nuclear fraction of Tead1. Altogether, their data, while not conclusive, are consistent with the notion that the localization of a TEAD protein may be influenced by interactions with one of its TIFs [55].

Two alleles of *sd*, *sd^68L^* and *sd^11L^*, have been mapped to the 3′ coding region of the gene. These alleles cause the lethal mutations Y355N and H433L, respectively [27]. The first causes a reduction in Vg nuclear localization in *sd^68L^* flies, even though the product of this mutant allele is able to interact with Vg *in vitro*. The second lies within the region deleted in Sd Δ421–440, which we have shown to be important for nuclear localization. Thus, we hypothesized that one or both of the regions altered in these mutants might be involved in the nuclear localization of Sd. However, both Sd^11L^ and Sd^68L^ are able to strongly direct an eGFP tag to the nucleus of S2 cells (data not shown). This implies that neither mutation directly impacts the nuclear localization of Sd. However, these results do reinforce the idea that the C-terminal domain has functions in addition to those already described.

In summary, data has been presented which indicates that the sub-cellular localization of Sd is dependent on multiple signals. The first is a bipartite cNLS. There is also evidence that suggests that an NES may be present as well. Furthermore, the domain C-terminal to the NES of Sd is important for the nuclear localization of the protein. While it seems likely that this is mediated by the ability of this domain to facilitate binding to cofactors, rather than direct binding to importins and exportins (although we cannot rule this possibility out), the mechanism by which this occurs is yet to be determined.

## Materials and Methods

### Construct design

Internal deletions were generated using inverse PCR followed by blunt-end ligation prior to cloning. Substitution mutations (mutations to the *sd* NLS coding sequence) were generated either by inverse PCR with non-overlapping primers, followed by blunt-end ligation prior to cloning, or by using inverse PCR with primers containing partially overlapping 5′ ends, followed by *Dpn*I treatment and transformation into *E. coli* (modified from [58]). Deletions, point mutations, the TEA coding sequence and the NLS coding sequence were cloned into pENTR using the pENTR/D-TOPO kit (Invitrogen Life Technologies). These constructs were then subsequently subcloned into pHGW (N-terminal eGFP), pHWG (C-terminal eGFP), pHFW (N-terminal 3xFLAG) or pTFW (N-terminal 3xFLAG, pUAST based transformation vector) using LRII recombinase (Invitrogen Life Technologies) according to the Murphy lab protocols (http://www.ciwemb.edu/labs/murphy/Gateway%20vectors.html#_References). In order to make C-terminal GFPx2-GST tagged proteins pMT/v5(A)+eGFPx2-GST was used (described in [42]. To clone into this vector, *Kpn*I restriction sites were appended to the NLS, TEA and the TEA ΔNLS coding domains using PCR amplification. These sites were then used for cloning 5′ to the tags. Oligonucleotides were used to append the palmitoylation, myristoylation and a linker domain to the *sd* coding sequence in order to generate PMSD, which was subsequently cloned in pENTR and subcloned into the monomeric red fluorescent protein (mRFP) tagging vector, pHRW. Oligonucleotides were also used to add the SV40 NLS coding sequence (which translates to PKKKRKV) into the *Not*I site of pENTR+Sd mNLS^N+C^. Routine PCRs were done with PlatinumTaq HIFI, while inverse PCRs were done with either Pfx^50^ or AccuPrime Pfx^50^ (all from Invitrogen Life Technologies). Primer details are available upon request.

### *Drosophila* Stocks

*Sd*, *PMSd-mRFP*, *Sd mNLS^N+C^* and *SV40-Sd mNLS^N+C^* were cloned into pTFW for subsequent micro-injection. The first was injected as described previously [59], into *y w*; *Δ2-3*/*Sb* embryos. The other two injections were performed commercially by BestGene (http://www.thebestgene.com/). At least two independent lines for each injection were generated. All crosses were performed at room temperature. *y w*; *Δ2-3*/*Sb* was a gift from A. Simmonds.

### Cell culture

S2 cells were obtained from Invitrogen Life Technologies. The cells were cultured in HyQ CCM3 (HyClone) at room temperature and 0.6 µg of the desired plasmids were transfected using Cellfectin (Invitrogen Life Technologies) according to the manufacturer's directions. In order to drive expression of GFP tagged constructs, the cells were heat-shocked @ 37°C for 40 minutes, approximately 36 h after transfection. pMT/v5(A) based constructs were induced by adding 0.4 mM CuSO_4_, 24 h after transfection. Induced cells were collected 38 hours post-transfection, washed, fixed in 2% paraformeldehyde and stained with DAPI diluted to a final concentration of 1 µg/ml. PBS was used as a buffer for all manipulations. The cells were mounted in PBS for imaging and coverslips sealed with VALAP (1∶1∶1 mixture of vasoline, lanolin and parafin wax [60]). For Leptomycin B treatment, cells were incubated with 25 nM of the chemical for 2 h prior to heat-shock.

Cells were imaged on a Zeiss 510 confocal microscope, using the appropriate filters for eGFP, mRFP and 4′,6-diamidino-2-phenylindole (DAPI). To minimize potential cross-talk between channels, scans were done sequentially. Images were initially imported and analyzed in ImageJ [61]. Subsequently Adobe Photoshop CS3 10.0 was used for final assembly (annotations and adjustments to brightness and contrast). Microsoft Excel 2007 was used to perform two-sample t-tests assuming unequal variance in order to test for statistical differences between the mean nuclear localizations.

Quantification of nuclear signal was done determining the total cellular signal and the nuclear signal using ImageJ. Cells were than normalized for both cytoplasmic and nuclear size. Finally, the normalized nuclear signal was divided by the normalized total signal to get the % nuclear signal. The % nuclear signal was then arbitrarily assigned to one of four categories: Nuclear denotes cells that contain exclusively or almost exclusively nuclear signal (>80% nuclear signal). Diffuse Nuclear includes cells which show predominant expression in the nucleus along with varying degrees of cytoplasmic signal (79–58% nuclear signal). Diffuse is for cells with signal approximately evenly distributed between the nucleus and cytoplasm or slightly enriched in the cytoplasm (57–36% nuclear signal). Excluded categorizes those cells which have exclusive or almost exclusive cytoplasmic signal (<35% nuclear signal).

### Co-immunoprecipitations

pHFW+Sd and pHFW+Sd mNLS^N+C^ were transiently transfected and induced in S2 cells as described above. A mock transfection was also done with water. Instead of fixing the cells, they were lysed in RIPA buffer (50 mM Tris-HCl, 150 mM NaCl, 1% NP-40, 0.5% deoxycholic acid, 0.1% SDS) containing Complete Protease Inhibitor Cocktail (Roche) for 15 min on ice. The lysed cells were then harvested and the lysate incubated with αFLAG M2 Affinity Gel (Sigma-Aldrich) for two hours at 4°C. The affinity beads were extracted and diluted into standard 4× SDS protein loading buffer. Equal amounts of 3xFLAG-Sd and 3xFLAG-Sd mNLS^N+C^ protein were loaded and separated on a 10% poly-acrylamide gel. Blotting was on Hybond ECL (GE Healthcare) with subsequent analysis using either anti-FLAG (Sigma-Aldrich) or anti-Importin-α3 [33] as primary antibodies. Detection was with horseradish peroxidase-labelled anti-mouse or anti-rabbit secondary antibodies (both at 1∶50000) and the SuperSignal Substrate Western Blotting kit (Pierce).

### Alignments

Jalview [62] was used to align TEAD containing sequences identified through BLASTp (www.ncbi.nlm.nih.gov, except EGL-44) searches using the Sd protein sequence as the query. EGL-44 was identified using wormbase (www.wormbase.org,WS204, July 29^th^ 2009).
